# Increased Levels of Inflammatory Cytokines and Endothelin-1 in Alveolar Macrophages from Patients with Chronic Heart Failure

**DOI:** 10.1371/journal.pone.0036815

**Published:** 2012-05-15

**Authors:** Liv I. Bjoner Sikkeland, Christen P. Dahl, Thor Ueland, Arne K. Andreassen, Einar Gude, Thor Edvardsen, Torbjørn Holm, Arne Yndestad, Lars Gullestad, Johny Kongerud, Pål Aukrust, Erik Øie

**Affiliations:** 1 Department of Respiratory Medicine, Oslo University Hospital Rikshospitalet, Oslo, Norway; 2 Department of Cardiology, Oslo University Hospital Rikshospitalet, Oslo, Norway; 3 Research Institute for Internal Medicine, Oslo University Hospital Rikshospitalet, Oslo, Norway; 4 Section of Clinical Immunology and Infectious Diseases, Oslo University Hospital Rikshospitalet, Oslo, Norway; 5 Department of Endocrinology, Oslo University Hospital Rikshospitalet, Oslo, Norway; 6 Faculty of Medicine, University of Oslo, Oslo, Norway; 7 Center for Heart Failure Research, University of Oslo, Oslo, Norway; National Institutes of Health, United States of America

## Abstract

**Background:**

Pathophysiological interactions between heart and lungs in heart failure (HF) are well recognized. We investigated whether expression of different factors known to be increased in the myocardium and/or the circulation in HF is also increased in alveolar macrophages in HF.

**Methodology/Principal Findings:**

Lung function, hemodynamic parameters, gene expression in alveolar macrophages, and plasma levels in the pulmonary and femoral arteries of HF patients (*n* = 20) were compared to control subjects (*n* = 16). Our principal findings were: (1) Lung function was significantly lower in HF patients compared to controls (*P*<0.05). (2) mRNA levels of ET-1, tumor necrosis factor (TNF)-α and interleukin-6 (IL-6) were increased in alveolar macrophages from HF patients. (3) Plasma levels of ET-1, TNFα, IL-6 and MCP-1 were significantly increased in HF patients, whereas our data indicate a net pulmonary release of MCP-1 into the circulation in HF.

**Conclusions/Significance:**

Several important cytokines and ET-1 are induced in alveolar macrophages in human HF. Further studies should clarify whether increased synthesis of these factors affects pulmonary remodeling and, directly or indirectly, adversely affects the failing myocardium.

## Introduction

Chronic heart failure (HF) is an important and increasing cause of cardiovascular morbidity and mortality. Despite the introduction of new therapeutic options, the five years mortality is above 50% [1], suggesting that important pathogenic mechanisms remain unchallenged by current treatment modalities. In patients with HF, the respiratory system has important clinical and prognostic implications. Although pathophysiological interactions between the heart and the lungs in HF are well recognized, most attention has been drawn to the effects of impaired left ventricular (LV) filling with increased filling pressure on pulmonary venous circulation [2], [3]. However, vasoactive peptides and cytokines known to be synthesized in the heart in HF may directly or indirectly affect the lungs and potentially also *vice versa.*

Activated alveolar macrophages synthesize numerous vasoactive, mitogenic and inflammatory mediators [4], and these cells are known to be important in various pulmonary disorders like chronic obstructive pulmonary disease (COPD) and sarcoidosis [5], . Previously, we have reported recruitment of macrophages into the pulmonary alveoli as well as increased expression of endothelin-1 (ET-1) [7], connective tissue growth factor (CTGF) [8] and adrenomedullin [9] in alveolar macrophages in experimental HF. Furthermore, previous investigations have suggested that recruitment of monocytes from the bloodstream to the lungs and alveoli is facilitated by cytokines, chemokines and vasoactive peptides [4], and several of these mediators are known to be induced in HF [10]–[12]. However, whether clinical HF is associated with activation of alveolar macrophages is not clear.

Increased synthesis and release of vasoactive, mitogenic and inflammatory mediators from alveolar macrophages in HF may have a local effect contributing to pulmonary inflammation and respiratory symptoms. Additionally, the release of these mediators could potentially also, directly or indirectly, have effects on the heart and the vessels. To further elucidate this issue, we in the present study examined the expression of various cytokines, chemokines and a vasoactive peptide known to be elevated within the failing myocardium and in the circulation in HF in alveolar macrophages from HF patients and healthy controls. We also related the expression pattern of these mediators to clinical, respiratory and hemodynamic characteristics of the HF patients.

## Materials and Methods

### Study Subjects

Twenty patients, age 53±10 years, with stable symptomatic HF for >3 months with NYHA class II-IV and LVEF <50% hospitalized at our hospital for evaluation of the etiology of HF or assessing whether they were candidates for cardiac resynchronization therapy or heart transplantation were consecutively included in the study. Patients with infections within the last 4 weeks, immunologic or connective tissue disease, and patients on anti-inflammatory medications were excluded. Also patients with spirometric values indicating COPD [forced expiratory volume in 1 second (FEV_1_)/forced vital capacity (FVC) <70%] were also excluded. None of the patients had known pulmonary disorders. Current or recent (within the last year before inclusion) smokers were also excluded. Thirteen patients were prior smokers, and eight of these patients had not been smoking for the last 15 years. Patient characteristics are shown in Table 1. Sputum and venous blood for RNA analysis (using PAXgene™ Blood RNA Tubes, PreAnalytiX, Hombrechtikon, Switzerland) were obtained from all HF patients, whereas right heart catheterization with blood sampling from the pulmonary artery and the femoral artery were performed in 15 of the patients. Prior to sputum induction, spirometry was performed using the Vmax system (Sensormedics, Yorba Linda, CA, USA) and in accordance with the guidelines recommended by the European Respiratory Society and American Thoracic Society [13]. Measurement of fractional eNO was performed at flow rate to a constant 50 mL/sec (NIOX, Aerocrine AB, Sweden). All patients were evaluated with echocardiography according to the recommendations of the American Society of Echocardiography [14]. Two control groups were included: 1) Ten non-smoking patients, age 47±8 years, undergoing right heart catheterization for electrophysiological examination served as controls for the plasma measurements in the pulmonary and femoral arteries. All of these patients had supraventricular arrhythmias but otherwise normal hemodynamic function without signs or symptoms of myocardial dysfunction. 2) Sixteen healthy non-smoking individuals, age 46±7 years, served as controls for examination of alveolar macrophages obtained from sputum and mRNA levels in peripheral blood cells.

**Table 1:** Clinical and hemodynamic characteristics of the HF population.

	HF patients (*n* = 20)
Age (year)	53±10
Gender (male/female)	19/1
Etiology (CAD/DCM)	10/10
NYHA class (II/III/IV) (%)	37/54/9
History (%)	
Diabetes mellitus	35
Hypertension	35
Previous myocardial infarction	50
Biochemical values	
Creatinine (µmol/L)	111±34
Nt-proBNP (pmol/L)	297±300
Hemodynamics	
LVEF (%)	29±9
PCWP (mmHg)	13±6
Cardiac index (L·min^–1^·m^–2^)	2.3±0.7
MPAP (mmHg)	24±11
PVR (dyne·s·cm^–5^)	191±143
Medication (%)	
ACE inhibitor	80
ARB	15
β-blocker	100
Diuretics	70
Aldosterone antagonist	55
Digitoxin	45
Statins	45
Warfarin	50

Data are presented as the mean ± SD or number or percentage of subjects. CAD, coronary artery disease; DCM, dilated cardiomyopathy; LVEF, left ventricular ejection fraction; PCWP, pulmonary capillary wedge pressure; MPAP, mean pulmonary artery pressure; PVR, pulmonary vascular resistance; ACE, angiotensin converting enzyme; ARB, angiotensin II receptor blocker.

The investigation conforms to the principles outlined in the Declaration of Helsinki. The study was approved by the regional ethical committee (Regional Committee for Medical Research Ethics South-East (REK Sør-Øst) and written informed consent was received from all participants.

### Right Heart Catheterization

Right heart catheterization was performed using a Swan-Ganz pulmonary artery thermodilution catheter (Baxter Health Care Corp., Santa Anna, CA, USA), and right atrial pressures, pulmonary artery pressures, and pulmonary capillary wedge pressure were measured. Cardiac output (CO) was recorded by thermodilution as a mean of 3–5 measurements and cardiac index calculated using CO divided by body surface area.

### Induced Sputum and Isolation of Alveolar Macrophages

Alveolar macrophages were collected from induced sputum. Three % (w/V), 4%, and 5% hypertonic saline were inhaled from an ultrasonic nebuliser (DeVilbiss, Sunrise Medicals, Longmont, CO, USA) during three 7-minute inhalation periods. At the end of each inhalation period, expectorates were collected and subsequently processed as previously described [15]. Possible contaminating HLA-DR-positive lymphocytes in induced sputum were removed using magnetic microbeads (Dynabeads Panmouse IgG; Invitrogen, Oslo, Norway) coated with CD3 antibody (Diatec Monoclonals AS, Oslo, Norway), before positive selection of alveolar macrophages by use of magnetic microbeads (Dynabeads Panmouse IgG; Invitrogen) coated with HLA-DR antibody (Diatec) [16]. Differential cell count of the beads selection showed a cell purity of 99% (median; range 96%–100%) macrophages, with contamination of mainly squamous cells. In each step the beads were incubated with the cells rotating for 10 minutes at 2–8°C before positive cell collection. Differential cell counts (Diff-Quik, Medion Diagnostics GmBH, Düdingen, Germany) were performed on a minimum of 300 cells by two blinded readers.

### Stimulation of Alveolar Macrophages

Alveolar macrophages were obtained by induced sputum from healthy adult volunteers as described above. The cells (75×10^3^ cells/mL) were incubated in 96 well polystyrene TC-Treated Microplate (200 µL/well; Corning, Schiphol-Rijk, Netherlands) for 48 hours before stimulating with or without a toll-like receptor (TLR)-4 agonist (LPS) from *E. coli* 026:B6 [100 ng/mL; Sigma, St Louis, MO, USA]), a TLR2 agonist (Pam_3_Cys, 1 µg/mL; Sigma), isoproterenol (20 µM; Sigma) and IL-1β (5 ng/mL; R&D Systems, Minneapolis, MN, USA). After 5 hours and 24 hours, cell-free supernatant was collected and stored at −80°C until use. The cells were cultured in RPMI (E15-840) (PAA Laboratories GmbH, Austria) with L-glutamine (2 mM) and added penicillin (100 U/ml), streptomycin (100 µg/ml) and 10% (culture medium) or 2.5% (stimulation medium) heat-inactivated fetal bovine serum (A15-101) (PAA Laboratories GmbH).

### Real-time Quantitative Reverse-transcription Polymerase Chain Reaction (RT-PCR)

Total RNA was isolated from alveolar macrophages and the PAXgene Blood RNA tubes and treated with DNaseI (PreAnalytiX). cDNA was synthesized using High Capacity RNA-to-cDNA Master Mix (Applied Biosystems, Carlsbad, CA, USA). Relative quantification of mRNA levels was performed by real-time RT-PCR using 7500 Fast Real-Time PCR System (Applied Biosystems). The relative standard curve method was used to compute the relative gene expression data. The oligonucleotide primers for PCR and the TaqMan probes were designed by the use of PrimerExpress software (Applied Biosystems) using sequences available from the GeneBank database (primer sequences can be provided on request). 18S rRNA (TaqMan rRNA control reagents, Applied Biosystems) was used for normalization of target mRNA results. The data acquired were analyzed with the Sequence Detector software (Applied Biosystems).

### Enzyme Immunoassay (EIA)

Concentrations of MCP-1 and ET-1 were measured by EIAs obtained from R&D Systems. Concentrations of TNF-α and IL-6 were measured by ultrasensitive EIAs provided from Invitrogen (Camarillo, CA, USA). The intra- and inter-assay coefficient of variation were <10% for all assays.

### Statistical Analysis

All the data are presented as mean ± SEM except for clinical and hemodynamic characteristics which are presented as mean ± SD. For comparisons of two groups, the Mann-Whitney U test was used. For comparisons of more than 2 groups, the Kruskal-Wallis test was used. If the Kruskal-Wallis test revealed significant differences, subsequent pairwise analyses of individual group means were performed with the Mann-Whitney U test. For comparison of paired data, Wilcoxon’s signed-rank test was used. The correlation analyses were assessed by Spearman rank correlation test. Value of *P<*0.05 was considered to be statistically significant. Linear regression analyses were used to investigate whether cytokine and ET-1 levels were influenced by previous smoking, adjusted for age and years since cessation of smoking.

## Results

### Lung Function, Diffusion Capacity, and Cellular Content in Sputum

As shown in Table 2, lung function and diffusion capacity were significantly reduced in HF patients (*n* = 20) compared to controls (*n* = 16). There was no difference in exhaled nitric oxide (eNO) levels between the two groups. Table 2 shows cell counts in sputum. Although there was no significant difference in total cell count per mg sputum, there was a trend toward increased cell count in HF patients compared to healthy control subjects.

**Table 2:** Lung function, exhaled NO value and differential sputum cell count in the induced sputum population.

	Control subjects	HF patients
	(*n* = 16)	(*n* = 20)
FVC (L)	5.1±0.3	4.0±0.2*
FVC % predicted	112±4	90±4*
FEV_1_ (L)	3.9±0.2	3.1±0.2*
FEV_1_% predicted	104±3	87±4*
FEV_1_/FVC	77±2	77±1
DLCO SI-units	10.8±0.6	7.2±0.5*
DLCO % predicted	101±4	71±4*
FeNO (ppb)	21±2	21±3
Total cell count/mg sputum	1039±194	1639±470
Neutrohpils/mg sputum	309±62	872±316
Macrophages/mg sputum	728±144	713±176
Lymphocytes/mg sputum	1±0.4	3±1
Eosinophils/mg sputum	1±0.3	32±21
% Neutrophils	33±5	42±5
% Macrophages	67±5	57±6
% Lymphocytes	0±0.0	0±0.0
% Eosinophils	0±0.0	1±0.3

FEV_1_, forced expiratory volume in 1 second; FVC, forced vital capacity; DLCO, diffusion capacity for carbon monoxide; eNO, exhaled nitric oxide; ppb, parts per billion. Data are mean ± SEM,

* p<0.05 versus controls.

### Gene Expression in Alveolar Macrophages and in Peripheral Blood

**Figure 1:**
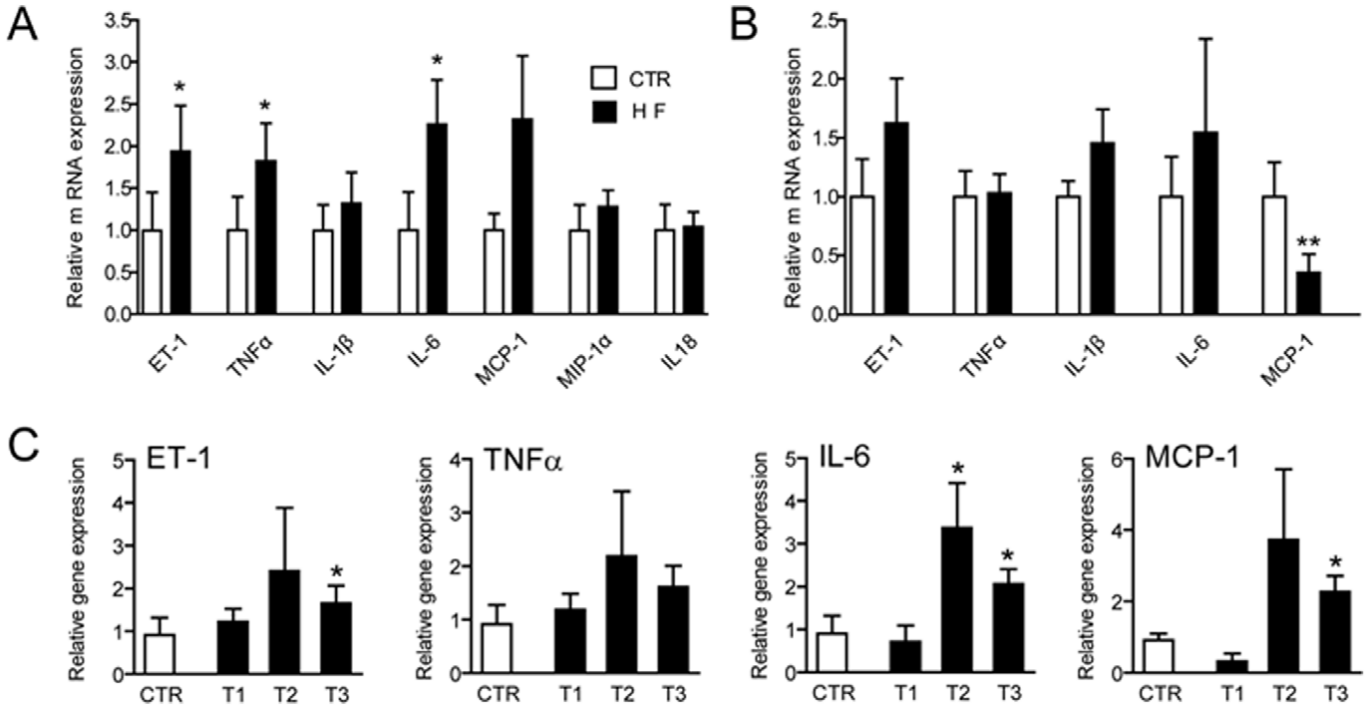
Gene expression in alveolar macrophages and in peripheral blood. A. mRNA levels of ET-1, TNFα, IL-1β, MCP-1, MIP-1α and IL-18 in alveolar macrophages in HF patients (*n* = 20) and controls (CTR, *n* = 16). B. Gene expression of the same mediators in peripheral blood cells. Gene expression was assessed by real-time quantitative RT-PCR in relation to the control gene18S rRNA. C. mRNA levels of ET-1, TNFα, IL-6 and MCP-1 in alveolar macrophages from HF patients (*n* = 20) and controls (CTR, *n* = 16) where mRNA levels in HF patients are separated into tertiles (T) according to left ventricular ejection fraction (LVEF) (T1, LVEF 34–47%; T2, 27–34%; T3, 20–25%). Gene expression was assessed by real-time quantitative RT-PCR in relation to the control gene18S rRNA. Data are mean±SEM. *p<0.05 and **p<0.01 versus controls.

As shown in Figure 1A, mRNA levels of ET-1, tumor necrosis factor (TNF)-α and interleukin (IL)-6 were significantly increased in alveolar macrophages from HF patients (*n* = 20) relative to controls (*n* = 16), but not gene expression of IL-1β, monocyte chemoattractant protein (MCP)-1, macrophage inflammatory peptide (MIP)-1α and IL-18. In contrast, mRNA levels of the mediators that were up-regulated in alveolar macrophages from HF patients did not show any increased expression in peripheral blood cells comparing HF patients and healthy controls (Figure 1B). Moreover, HF patients had decreased MCP-1 mRNA levels in peripheral blood as compared with healthy controls (Figure 1B). Prior smoking did not influence the cytokine or ET-1 levels in alveolar macrophages in a linear regression model adjusting for age and years since cessation of smoking.

### Correlation between mRNA Levels of Cytokines and ET-1 in Alveolar Macrophages and Clinical and Hemodynamic Parameters in HF Patients

When investigating the correlations between mRNA levels of ET-1 and the cytokines that were up-regulated in alveolar macrophages from HF patients (i.e., TNFα and IL-6) and clinical and hemodynamic parameters in these patients, we found a strong trend toward correlation between IL-6 mRNA levels and LV function [LV ejection fraction (EF); r = −0.53, p = 0.06]. When mRNA levels of the up-regulated mediators in alveolar macrophages from HF patients were separated into tertiles according to LVEF, we found a significant up-regulation of ET-1, IL-6 and MCP-1 in the tertile(s) with the lowest LVEF as compared with healthy controls (Figure 1C). In addition, IL-6 were positively correlated with the number of neutrophils in induced sputum (r = 0.87, p<0.001). There were no significant correlations between mRNA levels of the up-regulated cytokines in alveolar macrophages from HF patients and parameters of lung function and diffusion capacity in these patients (data not shown).

### Plasma Levels of Inflammatory Mediators in Pulmonary and Femoral Artery

**Figure 2:**
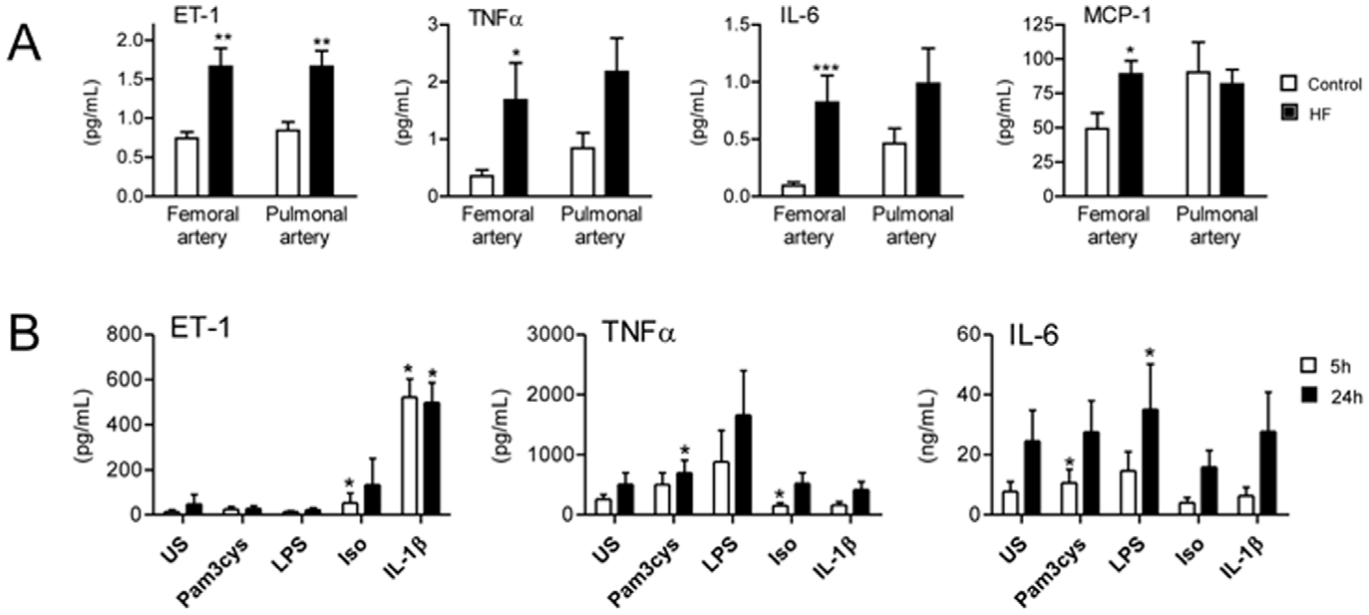
Levels of inflammatory mediators in pulmonary and femoral artery and after stimulation of alveolar macrophages. A. Plasma levels of ET-1, TNFα, IL-6 and MCP-1 in femoral and pulmonary artery in HF patients (*n* = 15) and controls (*n* = 10). Plasma levels of the various cytokines were measured by EIA. B. Release of ET-1, TNFα and IL-6 in alveolar macrophages from healthy controls (*n* = 5) that were cultured for 5 and 24 hours (h) with or without LPS (100 ng/mL), Pam_3_Cys (1 µg/mL), isoproterenol (20 µM) and IL-1β (5 ng/mL). Cytokine levels in supernatants were measured by EIA. Data are mean±SEM. *p<0.05, **p<0.01 and ***p<0.001 versus controls or unstimulated (US) cell, respectively.

As shown in Figure 2A, plasma levels of ET-1, TNFα, IL-6 and MCP-1 were markedly up-regulated in HF patients (*n* = 15) as compared with controls (*n* = 10) in the femoral artery. Increased plasma levels of ET-1 and a strong but not significant trend for increased plasma levels of TNFα and IL-6 were also found in the pulmonary artery. While there was no transpulmonary gradient for ET-1, TNFα and IL-6 in either HF or control patients, MCP-1 levels were higher in pulmonary as compared with femoral artery only in the controls, suggesting a net release of MCP-1 from the lungs during HF (p<0.05 when comparing transpulmonary gradients).

### Stimulation of Alveolar Macrophages

To elucidate the possible mechanisms for the up-regulated cytokines in alveolar macrophages from HF patients, we finally exposed alveolar macrophages from healthy controls to different stimuli with relevance to HF. As shown in Figure 2B, the TLR2 agonist Pam3Cys induced a significant increase in the release of IL-6 and TNFα from alveolar macrophages, whereas the TLR4 agonist lipopolysaccharide (LPS) induced a significant increase in the release of IL-6. In contrast to these rather modest effects, the β-receptor agonist isoproterenol and IL-1β induced a marked increase in the release of ET-1 (Figure 2B). None of the stimuli had any effects on MCP-1 release (data not shown).

## Discussion

In the present study, we show that patients with chronic HF have enhanced expression of several inflammatory and vasoactive mediators (i.e., ET-1, TNFα and IL-6) in alveolar macrophages as compared with healthy controls. Our findings suggest that chronic HF is accompanied by pulmonary inflammation involving activation of alveolar macrophages, potentially representing an inflammatory interaction between the lungs and the myocardium during HF.

It is well known that HF and COPD frequently coexist [17], [18]. In a recent study, the prevalence of COPD in a HF population, consisting of ∼70% previous or current smokers, was 35% based on spirometry-defined criteria [19]. In the current study of non-smoking HF patients, we found impaired lung function and diffusion capacity in HF patients as compared with healthy controls. Additionally, HF patients showed enhanced gene expression of ET-1, IL-6 and TNFα in alveolar macrophages as compared with healthy controls. A similar increase in mRNA levels was not found in peripheral blood, indicating that the increase in gene expression is primarily taking place in the lungs and is not a result of induction in monocytes in the circulation before entering the pulmonary compartment. We have previously shown enhanced expression of adrenomedullin in alveolar macrophages from HF patients [9], and our findings in the present study suggest that the activation of these cells during HF includes increased expression of several mediators with relation to HF.

Pulmonary remodeling is a well recognized consequence of chronic HF [20], but the mechanisms involved in this process are unclear. Previously, we reported enhanced pulmonary expression of CTGF and ET-1 in experimental HF, with enhanced expression in alveolar macrophages [7], [8], potentially contributing to pulmonary fibrosis and remodeling. In the present study, we show enhanced expression of ET-1 as well as several inflammatory cytokines in alveolar macrophages in clinical HF. In addition, IL-6 was correlated with the number of neutrophils in induced sputum. Based on the ability of these mediators to promote inflammation and vascular smooth muscle cell activation, it is tempting to hypothesize that the enhanced expression of these mediators could contribute to inflammation and pulmonary remodeling during HF. Moreover, HF patients had increased plasma levels of TNFα, IL-6 and ET-1 in both pulmonary and femoral artery as compared with controls, which indicate that both the lungs and the myocardium could be exposed to these mediators. However, although the lack of transpulmonary gradients, representing the net result of trapping and release of these mediators during pulmonary and myocardial circulation, does not exclude an inflammatory interaction between the lungs and myocardium in HF, the hypothesis that this inflammatory loop could contribute to remodeling in both organs will have to be examined in forthcoming studies.

The reason for the enhanced expression of several inflammatory mediators in alveolar macrophages during HF is at present not clear, but could involve mechanisms such as pulmonary vascular congestion and shear stress secondary to chronic HF. Also, it is well known that pulmonary bacterial infections could induce exacerbation of cardiovascular disease [21]. This phenomenon could potentially involve TLR-mediated activation of alveolar macrophages, and in the present study we show that TLR2 and TLR4 activation induce IL-6 and TNFα release in these cells. Moreover, ET-1 is a potent inflammatory and vasoactive cytokine that has been suggested to participate in the pathogenesis of myocardial failure and pulmonary remodeling during HF [22], [23]. Here, we show that IL-1β, representing an upstream inflammatory mediator in innate immune responses, and β-adrenergic receptor activation profoundly induce the release of ET-1 from alveolar macrophages. ET-1 has been shown to induce IL-1β production [24], and if such interactions are operating in alveolar macrophages, it could represent an inflammatory loop that is operating in the lungs during HF.

In conclusion, we found that several important cytokines are induced in alveolar macrophages in human HF. However, relatively few patients were examined and the potential pathogenic importance of alveolar macrophage-mediated inflammation in HF is at present unclear. Further studies should clarify whether this induction affects pulmonary remodeling and whether the increased synthesis of these factors, directly or indirectly, may adversely affect the failing myocardium.
